# Binding of Factor VIII to Lipid Nanodiscs Increases its Clotting Function in a Mouse Model of Hemophilia A

**DOI:** 10.4172/2155-9864.1000325

**Published:** 2015-12-18

**Authors:** Keri Csencsits-Smith, Krill Grushin, Svetla Stoilova-McPhie

**Affiliations:** 1 Department of Pathology and Laboratory Medicine, University of Texas Health Science Center, Houston, TX-77030, USA; 2 Department of Neuroscience and Cell Biology, University of Texas Medical Branch, Galveston, TX-77555, USA

**Keywords:** Factor VIII, Lipid nanodiscs, Hemophilia A, FVIII deficient mice, Drug delivery

## Abstract

**Background:**

Hemophilia A is a congenital bleeding disorder caused by defective or deficient factor VIII (FVIII). The active form of FVIII is the co-factor for the serine protease factor IXa (FIXa) in the membrane-bound intrinsic tenase (FVIIIa-FIXa) complex. The assembly of the FVIIIa-FIXa complex on the activated platelet surface is critical for successful blood clotting.

**Objectives:**

To characterize the role of lipid nanodiscs (ND) for on FVIII function in vivo and test the lipid ND as a delivery system for FVIII. To evaluate the potential of binding recombinant FVIII to ND as improved treatment for Hemophilia A.

**Methods:**

Recombinant porcine FVIII (rpFVIII) was expressed and characterized in solution, and when bound to ND. The rpFVIII, ND and rpFVIII-ND complexes were characterized via transmission electron microscopy. Functional studies were carried out using aPTT tests and time resolved tail snip studies of hemophilic mice.

**Results:**

Functional rpFVIII was successfully assembled on lipid ND. When injected in hemophilic mice, the rpFVIII-ND complexes showed a pronounced pro-coagulant effect, which was stronger than that of rpFVIII alone. While injection of the ND alone showed a pro-coagulant effect this effect was not additive, implying that the rpFVIII-ND complexes have a synergistic effect on the clotting process in hemophilic mice.

**Conclusions:**

Binding of rpFVIII to ND prior to its injection in hemophilic mice significantly improves the therapeutic function of the protein. This represents a meaningful step towards a new approach to modulate blood coagulation at the membrane-bound FVIII level and the assembly of the intrinsic tenase complex.

## Introduction

**Figure 1:**
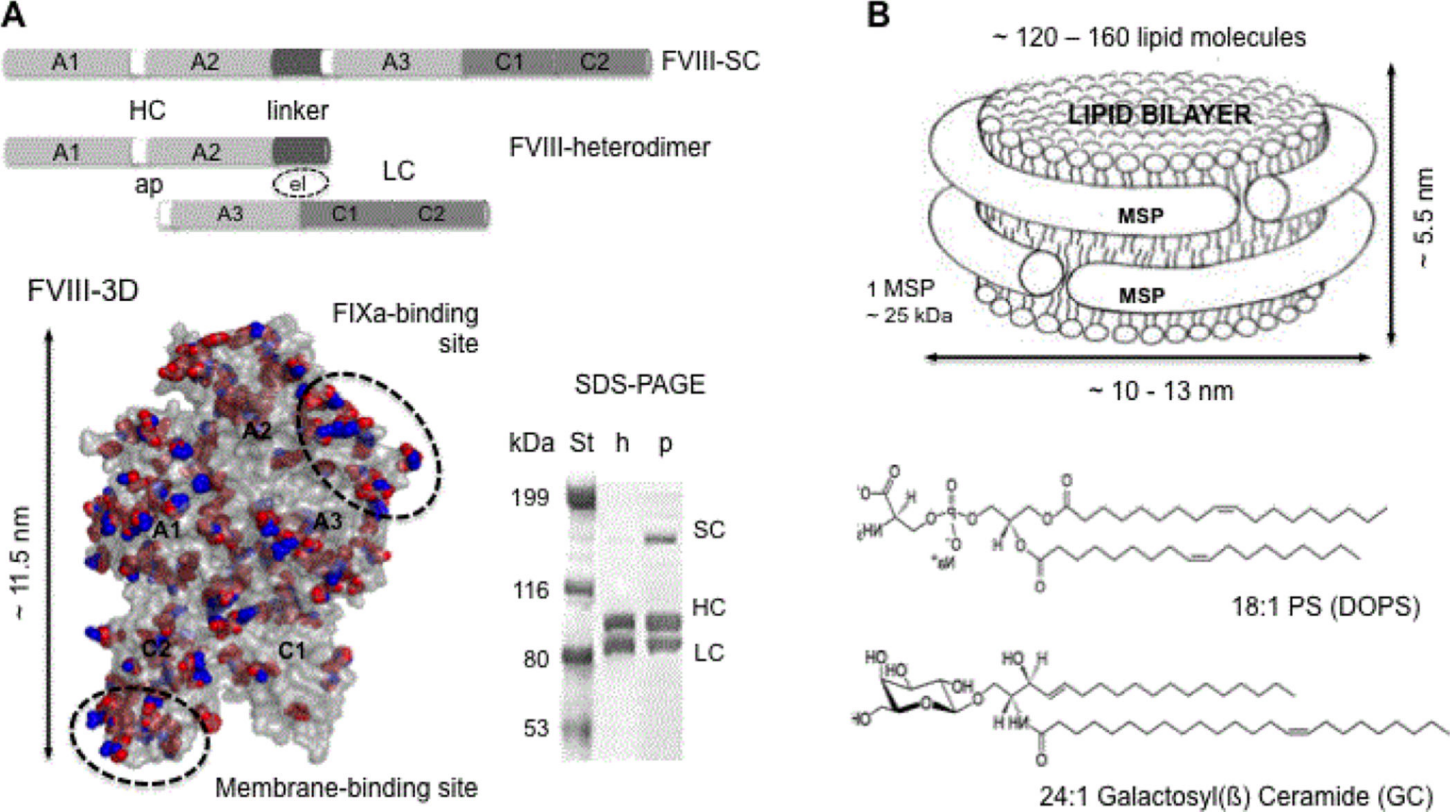
A. Domain organization of recombinant human (h) and porcine (p) FVIII lacking the B domain. The heavy (HC) and light (LC) chains are linked with a peptide of 24 amino acid (aa) residues for the porcine and 21 aa residues for the human FVIII single chain (SC) forms. The HC and LC are linked electrostatically (el) in the FVIII heterodimer, which is the predominant form of FVIII *in vitro*, as shown on the SDS-polyacrylamide gel (PAGE). In white are shown the activation peptides (ap). The hFVIII crystal structure (FVIII-3D, 3CDZ.PDB) is shown as a grey surface. The side chains of the aa residues of the hFVIII, which differ from the pFVIII are shown as red spheres and the side chains of the aa residues of the pFVIII that differ from the hFVIII are shown as blue spheres. The FVIII domains are indicated, as well as the main membrane- and FIXa-binding sites. B. Schematic of a lipid nanodiscs (ND) circled by two membrane scaffolding proteins (MSP). The chemical structure of the lipids employed for the ND in this study: phosphatidylserine (PS) and galactosylceramide (GC) are also shown.

Hemophilia A is an X-linked recessive congenital disorder caused by defective or deficient FVIII [1]. The major treatment for Hemophilia A is the infusion of factor VIII (FVIII), which restores hemostasis [2]. Factor VIII is a large 300 kDa glycoprotein of 2332 amino acid residues expressed as six distinct domains termed A, B and C. These domains form two polypeptide chains: a heavy chain (HC ~ 90 – 110 kDa) of the A1-A2-B domains and a light chain (LC ~ 80 kDa) of the A3-C2-C2 domains [3,4]. The HC and LC are held together covalently by the B/linker domain as a single chai or associated or by non-covalent interactions following proteolytic cleavage of the B domain (Figure 1A) [4–6]. The A2 and A3 domains contain the main protease (FIXa) binding sites [5], while the C domains hold the main membrane-binding sites (Figure 1A) [7,8]. So far, the correlations between FVIII membrane binding and specific function are not fully understood [9–12].

After the FVIII gene was cloned in 1984, replacement therapies using recombinant FVIII products were introduced for the treatment of Hemophilia A [4,13,14]. Recombinant hFVIII (rhFVIII) concentrates have very similar biochemical, hemostatic and pharmacokinetic profiles to plasma-derived FVIII [15,16]. Continuing efforts to improve the safety of replacement therapies have led to a new generation of recombinant products that provide a normal or near-normal life expectancy for most people with Hemophilia A [17,18]. Recombinant human FVIII lacking the B domain is now the most common form used for the treatment of Hemophilia A. Removal of the B domain from the cDNA sequence (~38%) improves FVIII yield without loss of its pro-coagulant activity. Recombinant porcine FVIII lacking the B domain (rpFVIII) shares 86% amino acid sequence identity with rhFVIII, has similar coagulation activity and forms functional tenase complexes with human FIXa both *in vitro* and *in vivo* [19,20]. Expression of rpFVIII is increased 10–14 fold over its human homologue and its activated form, rpFVIIIa, is stable compared to rhFVIIIa, which spontaneously dissociates after a few minutes *in vitro* [21–23].

The management of Hemophilia A in patients requires frequent and multiple infusions of FVIII, due to its relatively short half-life (~12 hours when injected intravenously) [24]. Thus, there is ongoing research to develop rFVIII products with an extended half-life to improve FVIII potency and pharmacokinetic properties [12,25]. Novel FVIII constructs were obtained by direct chemical modification, such as PEGylation-covalently attaching low-molecular weight polyethylene glycol (PEG) to the rhFVIII, or by genetic engineering-fusing the rhFVIII molecule to the Fc fragment of human immunoglobulin G (IgG1) [13,18,24,26]. These strategies, however, do not address the loss of potency due to alterations in protein-protein and protein-membrane interactions important for FVIII function [13,18,24].

Another serious complication of Hemophilia A treatment is the development of inhibitory antibodies against rhFVIII concentrates, which affects ~30% of patients [27]. Alternative strategies have been developed to avoid the immune response to FVIII, such as bypassing the intrinsic tenase complex through the factor VII pathways (extrinsic tenase complex) and chemical modifications that reduce FVIII immunogenicity in vivo [13,18,24,26]. Porcine FVIII products have low immunogenicity and have been successfully introduced as therapeutic option for patients with Hemophilia A that develop inhibitory antibodies against human FVIII [19,28].

There is increasing interest in the application of lipid nanotechnology to improve therapeutic administration of pharmaceuticals. Nanodiscs (ND) are such lipid nanotechnologies, composed of a stable discoidal lipid bilayer encircled by amphipathic membrane scaffold proteins (MSP) that self-assemble in aqueous solutions [29,30]. These MSP are short alpha-helical recombinant membrane proteins that are an engineered variant of human Apolipoprotein A-I (APO-AI), the major protein component of high density lipoprotein (HDL) in blood plasma [31]. While HDL generally circulates as a spherical particle with cholesterol esters in its core, ND are discoidal in shape with a diameter of ~9–16 nm depending on the lipid composition, MSP to lipid ratio and the length of the MSP [32] (Figure 1B). The monodispersity and solubility of the ND have proven to be extremely useful for structural and functional studies of membrane and membrane-associated proteins at close to physiological conditions [30,33–35]. ND are suitable for stabilizing membrane-bound coagulation factors and complexes [32,36], and for applications in drug discovery [32,37].

In this report we present our novel results showing the potential of phosphatidylserine (PS) containing lipid ND for stabilizing blood coagulation FVIII and improving its clotting function in hemophilic mice.

## Materials and Method

### Materials

HEPES sodium salt, sodium cholate (Na-cholate) and MSP1D1 scaffolding protein were obtained from Sigma (Sigma-Aldrich, St. Louis, MO). Sodium chloride (NaCl) and calcium chloride (CaCl_2_) were obtained from J.T. Baker (Mallinckrodt Baker, Inc. Phillipsburg, NJ). Bio-Beads SM-2 were purchased from Bio-Rad Laboratories (Hercules, CA, USA). Phospholipids-DOPS (1,2-dioleoyl-sn-glycero-3-phospho-L-serine sodium salt) and GC (D-galactosyl-β-1,1' N-nervonoyl-D-erythro-sphingosine, d18:1/24:1(15Z)) were obtained from Avanti Polar Lipids, Inc (Alabaster, AL, USA). All buffer solutions – HBS (NaCl 150 mM, HEPES 20 mM, pH 7.4) and HBS-Ca^2+^ (NaCl 150 mM, HEPES 20 mM, CaCl_2_ 5 mM, pH 7.4) were filtered through 0.22 µm Millex^®^ GP filter (Millipore, Carrigtwohill, Co. Cork, Ireland).

### Expression and purification of porcine Factor VIII (rpFVIII)

B-domain deleted porcine FVIII (rpFVIII OL, POL1212) was expressed in BHK cells provided by Professor Lollar’s laboratory (Emory University, Atlanta, GA) and purified following established protocols [32,38]. The fractions containing rpFVIII were combined, buffer exchanged against HBS-Ca buffer and concentrated through a 0.22 µm Millex^®^ GP filter (Millipore, Carrigtwohill, Co. Cork, Ireland). The protein concentration was estimated with a Nanodrop Spectrophotometer (ND-1000 ThermoFisher Sci, Waltham, MA) [32].

### SDS-Polyacrilamide gel electrophoresis (PAGE)

Ready Gel^®^ Tris-HCl Gels (4–15%) (Bio-Rad, USA) were run according to standard protocol [20]. The rpFVIII sample at 2 µg/well concentration was reduced with 5% β-mercaptoethanol in Bio-Rad Laemmli Sample buffer at 94°C for 5 min, loaded onto gels with Bio-Rad Pre-stained Standards Broad Range and run at 115 V for 2 hours 30 minutes. The gels were stained with GelCode^®^ Blue Stain Reagent (Thermo Scientific, USA) and de-stained in water.

### Assembly and purification of phosphatidylserine (PS) rich nanodiscs (ND)

ND were prepared following previously established protocol [32,34]. Briefly, MSP1D1 was lyophilized in HBS buffer containing 15 mM Na-cholate. The phosphatidylserine (PS) and Galactozylceramide (GC) lipids were dissolved in chloroform and mixed at 1:4 weight ratio. Chloroform was evaporated under argon gas and the lipid residue lyophilized in HBS buffer containing 26 mM Na-cholate, warmed up to 70°C and sonicated for 15 min. The MSP1D1 and lipids were mixed at 47:1 lipid to protein ratio and incubated for 1 hour at 37°C. The Na-cholate was removed by adding 1g Bio-Beads SM-2 per 1 ml of sample and incubated for 4 hours at room temperature. The Bio-Beads were removed by centrifugation and the ND suspension further purified by fast protein liquid chromatography (FPLC) through a Superdex 200 HR 10/30 column equilibrated in HBS. The fraction corresponding to ND ~12 nm diameter was pooled and concentrated to 1 mg/ml in a Vivaspin with a 10 k cutoff membrane, as previously described [32].

### Assays of rpFVIII and rpFVIII-ND activity

The activity of rpFVIII and the rpFVIII-ND complexes was evaluated with the one-stage activated partial thromboplastin time clotting test (aPTT) using a Diagnostica Stago clotting instrument, as previously described [32,38]. Samples were incubated in human FVIII deficient plasma (<1% activity, George King Bio-Medical, Inc.) with aPTT reagent (TriniCLOT Automated aPTT, Tcoag Ireland Limited, Ireland) for 4 min at 37°C. The FVIII activity was evaluated against Factor Assay Control Plasma (FACT, 1 U/ml of FVIII activity; George King Bio-Medical, Inc.).

### Negatively stained electron microscopy (NS-EM) of rpFVIII, ND and rpFVIII-ND complexes

The ND were diluted to 0.005 mg/ml based on the MSP1D1 protein concentration. 5 µl of the sample was deposited onto a freshly carbon coated and plasma-treated hexagonal copper EM grid (300 mesh, Ted Pella, Inc., CA, USA) and stained with 1% Uranyl acetate aqueous solution. The rpFVIII-ND complexes were prepared by mixing the initial rpFVIII and ND solutions (at 1 mg/ml concentration each) at 1:1 weight ratio. After 5 min incubation at room temperature, the samples were diluted to a final concentration of 0.002 mg/ml and stained with 1% Uranyl acetate. Digital electron micrographs were collected with a JEM1400-LaB6 transmission electron microscope (JEOL, Ltd) operated at 120 kV with a 2048 × 2048 pixel Ultrascan CCD camera at a final magnification of 84,000×.

### Time resolved snip tail tests of rpFVIII, ND and rpFVIII-ND complexes injected in hemophilic (FVIII deficient, FVIII-KO) mice

FVIII deficient mice, exon 16 deleted mice backcrossed onto the C57BL/6 mouse strain, were kindly provided by Dr. David Lillicrap (Queens University, Ontario, Canada). This mouse model is known for having a robust T_H_1 and T_H_2 inflammatory cytokine response and produces high antibody titers in response to FVIII injection [39]. A breeding colony was established and maintained in the Center for Laboratory Animal Medicine and Care facility at the University of Texas Health Science Center-Houston under an Animal Welfare Committee approved protocol. Mice were housed in IVC (Individually Ventilated Cages, Tecniplast, Buguggiate, VA, Italy) under pathogen-free conditions and fed sterile food and water ad libitum.

Stock rpFVIII and ND (at 1 mg/ml concentrations) were mixed at 1:1 weight ratio in HBS-Ca buffer. Prior to injection, the rpFVIII, ND and rpFVIII-ND solutions were diluted to 0.084 mg/ml. The mice were anesthetized to effect (3–5 minutes) with isoflurane in an induction chamber, then retro-orbital injection [40] with of 50 µl diluted rpFVIII, ND or rpFVIII-ND (4.2 µg rpFVIII or ND, respectively) was performed. The mice were rested for 15 min then anesthetized to effect. Tails were snipped 5 mm distal and blood loss was measured by blotting the tail for 10 seconds against a piece of hardened and ashless Whatman filter paper #540 (Sigma-Aldrich). This procedure was repeated every 30 seconds until clotting occurred up to 20 minutes. Following anesthesia to effect, bleeding was stopped using a hand-held cautery pen (Jorgenson Industries Inc., Loveland, CO). For the control mice the tail snip was performed following anesthesia to effect.

### Immunization of the hemophilic (FVIII-KO) mice with rpFVIII, ND, or rpFVIII-ND

2 µg rpFVIII, ND, or ND-rpFVIII in 50 µl sterile PBS was retro-orbitally injected every 7 days for a total of 4 injections. Mice were euthanized and blood samples were collected 3 days following the final injection. Blood samples were collected in 10% sodium citrate/blood volume, centrifuged at 1400 rpm for 20 minutes, and plasma was stored at −80°C until use.

### Anti-FVIII ELISA

Flat-bottom, medium-binding, Microlon 96-well ELISA plates (Greiner Bio-One, Monroe, NC) were coated with 1 µg/ml rpFVIII dissolved in 100 mM NaHCO_3_, pH 9.5 and incubated at 37°C for 1 hour. The plate was then blocked with 5% skim milk dissolved in 0.05% PBS/Tween 20 (PBS-T; Sigma) at 37°C for 1 hour. The mouse plasma samples were serially diluted 1:2, starting at a 24 dilution, in 1% skim milk dissolved in 0.05% PBS/T and incubated at 37°C for 2 hours. The plate was then incubated with a 1:1000 dilution of goat anti-mouse-IgG Fc specific horseradish peroxidase (HRP) conjugated antibody (Sigma; A2554) and incubated at 37°C for 1 hour. The HRP was detected by addition of TMB-US substrate (Moss, Inc. Pasadena, MD) and allowed to react at room temperature in the dark for 20 minutes. The reaction was stopped by the addition of 2N H_2_SO_4_. Absorbance was determined at 490 nm on a Bio-Rad 3550 plate reader. Endpoint titers were defined as the highest serial dilution that resulted in a reading of 0.2 OD over background.

## Results

### Characterization of rpFVIII in solution and when bound to PS enriched lipid ND

Fully functional recombinant porcine FVIII lacking the B domain (rpFVIII) was expressed in sufficient quantity for the in vivo tests (Figure 1) [20]. The activity of rpFVIII in solution and when bound to PS-ND was tested with the one stage aPTT assay against FVIII deficient human plasma and in the presence of 10:1 excess of ND (Table 1). The slight increase in activity observed for the membrane-bound rpFVIII to ND can be explained by the stabilizing effect of the membrane. The ND alone did not show any pro-coagulant activity in FVIII deficient plasma.

**Figure 2:**
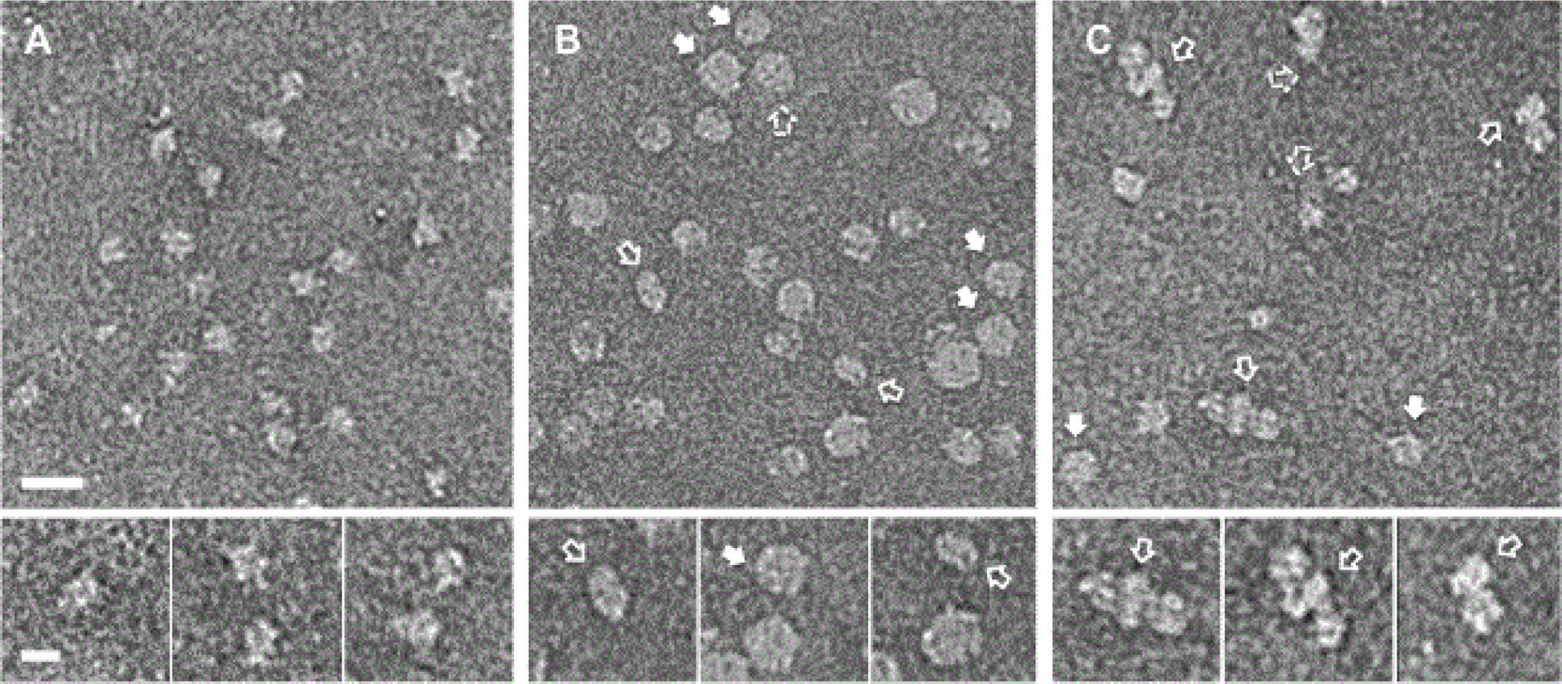
Electron micrographs of negatively stained A. Recombinant pFVIII molecules. B. Lipid nanodiscs (ND) containing phosphatidylserine and C. Recombinant [FVIII molecules attached to the lipid ND in B. The full arrows show top and bottom views of the ND and rpFVIII-ND, respectively. The empty arrows show the side views of the ND and the dashed arrows show tilted views of the ND and rpFVIII-ND. The scale bar is 20 nm. The insets show magnified views of the rpFVIII, ND and rpFVIII-ND complexes. The scale bar is 10 nm.

The rpFVIII was homogeneous in solution, as previously described [20,32]. Electron microscopy (EM) of negatively stained rpFVIII molecules adsorbed on amorphous carbon film showed predominantly monomeric distribution, as previously observed with cryo-electron microscopy [12] (Figure 2A). The fraction of the ND employed for the in vivo studies was assembled at 1:47 MSP1D1 to lipid ratio and at 80% PS. The ND showed two main size distributions: ~12 nm and ~15 nm diameters, which confirmed our previous EM and dynamic light scattering studies (Figure 2B) [32]. The rpFVIII molecules were clearly visible in the EM micrographs when bound to the ND, and were more often attached to both sides of the ND membrane [32]. No significant rpFVIII-ND aggregation was observed under these experimental conditions (Figure 2C).

### Measurement of the rpFVIII, ND and rpFVIII effect on clotting in hemophilic (FVIII-KO) mice

**Figure 3:**
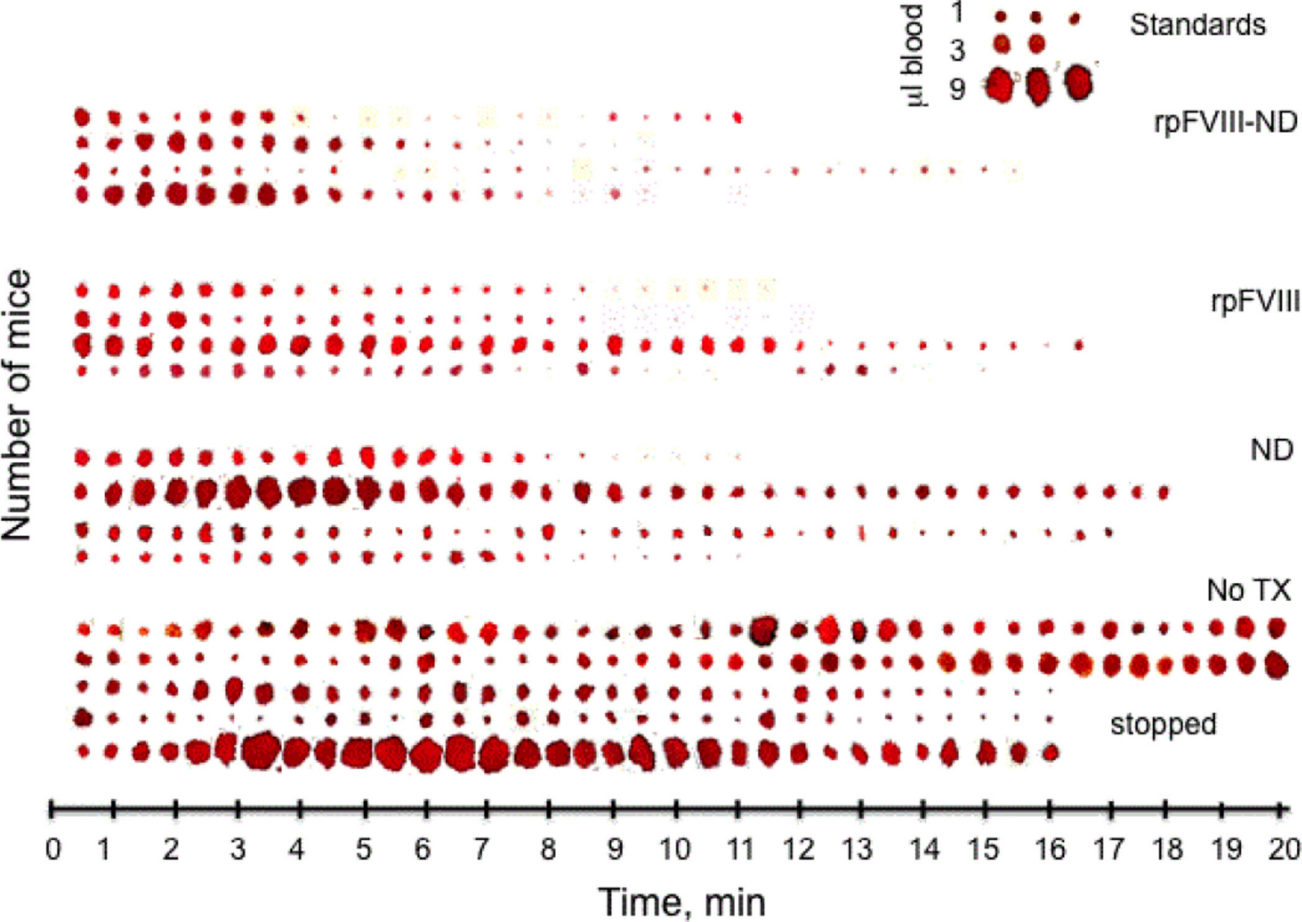
Tail-snip tests of hemophilic (FVIII-KO) mice measured over 20 minutes after administrating of lipid nanodiscs (ND), rpFVIII and rpFVIII bund to ND (rpFVIII-ND). No TX is no treatment. The snipped tails of the mice were blotted each 30 seconds for 10 seconds starting form the time (0) of the snip. Each ‘blood spot’ corresponds to the amount of blood blotted to a hardened filter paper. The filter papers with the blood spots were digitized, cropped and plotted against the time line. The inset shows drops of mice blood with a known volume in microliters (µl), blotted against the same filter paper used for the tail-snip tests.

**Figure 4:**
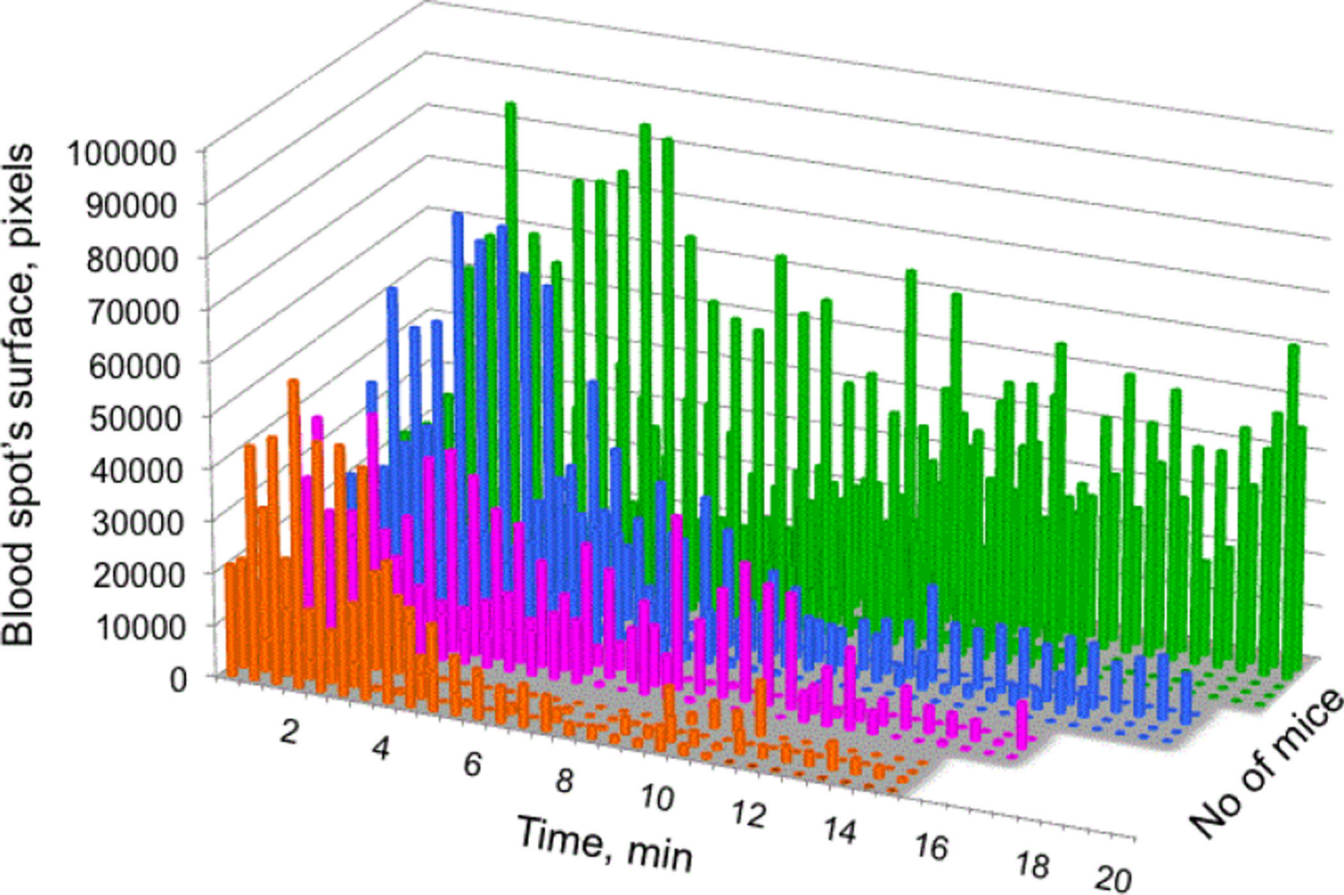
Time course from the tail-snip tests of hemophilic (FVIIIKO) mice after administrating of: lipid ND - blue, rpFVIII - magenta, rpFVIII bound to ND (rpFVIII-ND) - orange and no treatment (no Tx) in green. The time course is shown as a graph of the surface of the ‘blood spots’ from Figure 3 in arbitrary pixels for each mouse over 20 minutes.

**Figure 5:**
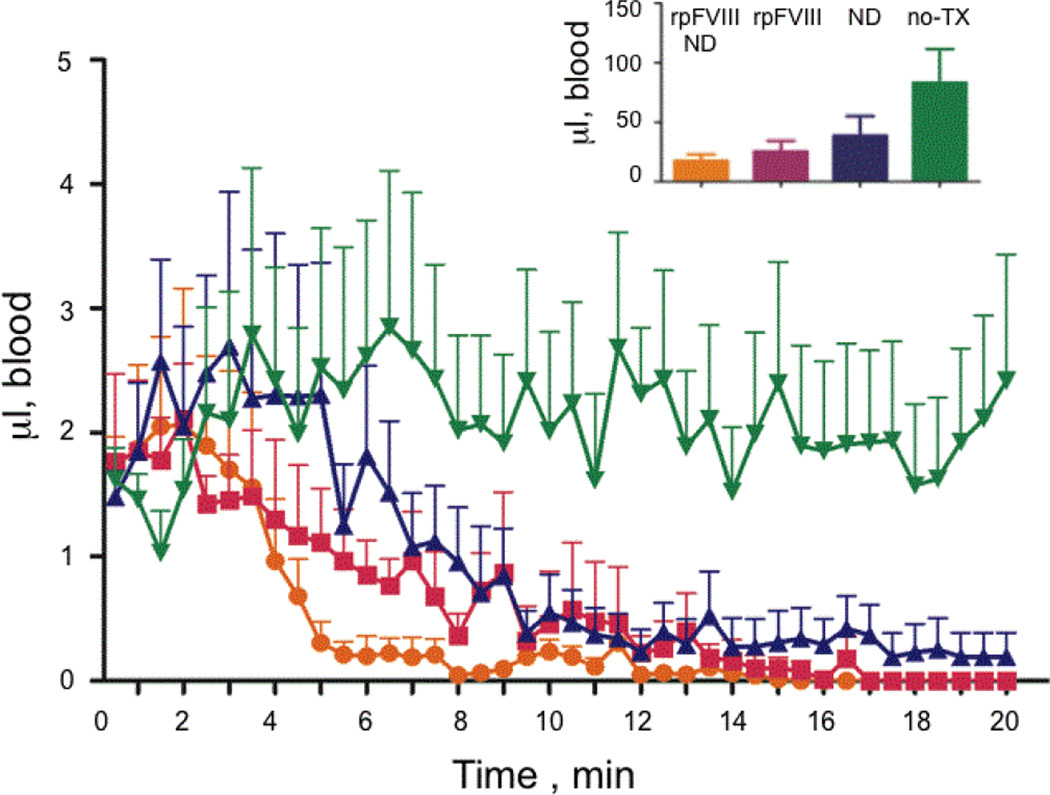
Time resolved bleeding of Hemophilia A mice after injection of ND (blue), rpFVIII (magenta), rpFVIII-ND (orange) and no treatment (no-TX, green). The averaged values of the data presented in Figure 4 are shown over 20 minutes. The inset shows the averaged total volumes of the bleeding after injection and tail snip over 20 minutes. The standard deviation of each time point and the total volumes are shown as bars.

To test the effect of ND on the FVIII function in vivo, we measured the clotting time following injection of rpFVIII, ND or rpFVIII-ND solutions in hemophilic (FVIII-KO) mice. To assess the time course of clotting, we designed a method to measure blood loss by blotting the tip of the mouse tail after the tail snip against hardened and ashless filter paper for 10 seconds, repeated every 30 seconds over a total time of 20 minutes (Figure 3). We ensured that tails were cut at the same angle and distance from the tip, and blotted to the filter paper in a consistent manner. A similar method of measurement was recently reported to be an accurate assessment of clotting activity in the FVIII-KO mice [41]. The filter papers were digitally scanned and the size of blood spots was plotted against time of collection (Figure 3). The scanned and digitized filter papers were then converted into HDF format using EMAN2 software for Image analysis [42] and imported into the UCSF Chimera suite for interactive visualization and analysis [43]. The surface area of the ‘blood spots’ was determined in arbitrary pixels using the USCF Chimera ‘color and measure blobs’ option, and plotted against time (Figure 4). The surface areas of the ‘blood spots’ were further calibrated against spots obtained using known blood volumes of 1, 3 and 9 µl (Figure 3, inset). The calculated average volumes corresponding to the blood loss were plotted against time (Figure 5). A two-way statistical analysis was applied using analysis of variance (ANOVA) as implemented in GraphPad Prism 6.0 (GraphPad Software Inc., La Jolla, CA, USA) to determine the differences between the rates of clotting among the treatment groups. We observed significant heterogeneity in the clotting time curves following tail snips, as previously reported [44,45]. This heterogeneity was more pronounced for the control mice (which received no treatment) and mice injected with ND alone (Figure 5).

Our results show that binding of rpFVIII to ND significantly improves rpFVIII pro-coagulant properties in vivo. Injection of ND without bound FVIII showed a pro-coagulant effect, which was not detected in the aPTT clotting assays *in vitro*. The clots that formed following the injection of ND solution, however, proved to be unstable and detached easily following blotting on the filter paper (Figure 4, blue). The bleeding rate and time to clot in the hemophilic mice that received rpFVIII bound to ND didn’t have an additive effect when compared to ND and rpFVIII alone, indicating that binding of rpFVIII to ND induced better and more stable clot formation over time (Figure 4 and 5). The deviation in the time to clot between the mice that received the rpFVIII-ND combination was less variable, suggesting a radical improvement of the therapeutic effect. Treatment with membrane-bound rpFVIII (rpFVIII-ND) also resulted in less total blood loss measured over 20 minutes than did treatment with rpFVIII in solution (Figure 5, inset). Finally, a statistical analysis of blood loss over time with NOVA revealed that the mice receiving rpFVIII-ND had a more rapid time to clot formation than did mice that received rpFVIII alone. All curves depicted in Figure 5 (orange pFVIII-ND, magenta rpFVIII and blue ND) are significantly different than the no-treatment (green) curve and all have a p value <0.0001. Calculation of total blood loss (Figure 5 inset) did not show significant difference between treatment groups (p values vs. control (green) for the rpFVIII-ND (orange)=0.1071, for the rpFVIII (magenta)=0.1002 and for the ND (blue)=0.2442).

### Immunogenicity of rpFVIII and rpFVIII-ND in FVIII-KO mice

**Figure 6:**
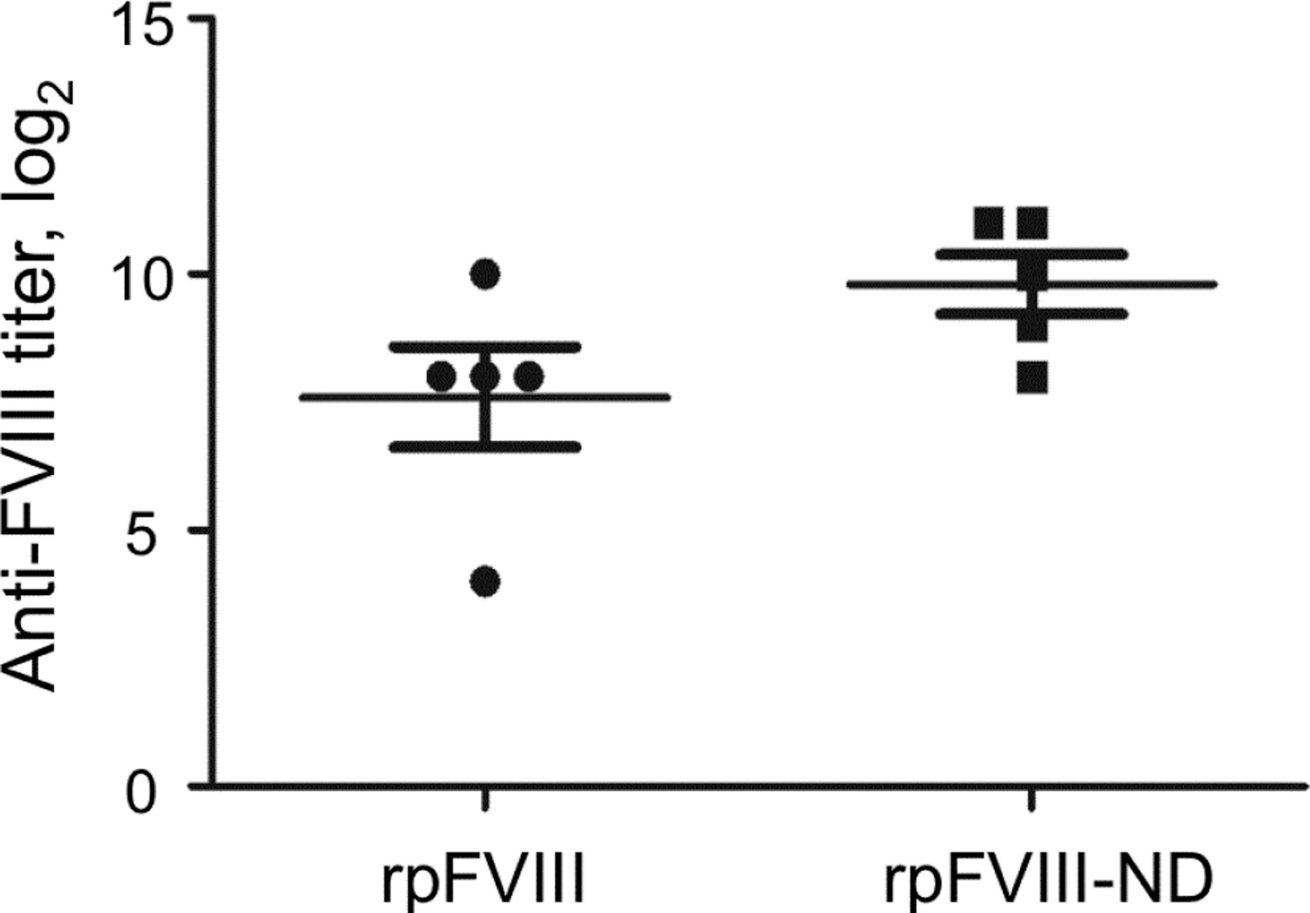
Anti-FVIII ELISA titer of rpFVIII (circles) and rpFVIII-ND (squares). FVIII deficient mice were injected every 7 days with 2 µg/mouse recombinant porcine FVIII alone or porcine FVIII-ND complex. Each mouse received 4 doses, and plasma was obtained one week after the final dose. Serial dilutions (1:2) of plasma were added to ELISA plates coated with porcine or human FVIII, and the highest plasma dilution that produced a reading of ≥0.2 OD over background at 450 nm were reported as endpoint titers.

To test if assembly of rpFVIII with ND affected its immunogenicity we injected FVIII-KO mice 4× with 2 µg/mouse of rpFVIII alone or rpFVIII ND. As shown in Figure 6, the injection of combined rpFVIII-ND resulted in slightly, but not significantly (p=0.090) increased plasma levels of rpFVIII specific IgG antibody (average titer of 2^8^ v/s 2^10^). The anti-FVIII titers produced in FVIII-KO mice in response to injection of rpFVIII-ND complexes were comparable to those reported in other studies following repeated doses of recombinant FVIII [46,47].

## Discussion

The rationale to test the effect of membrane binding on the rpFVIII clotting efficiency in hemophilic mice was to investigate i) whether binding of rpFVIII to ND could improve clotting time compared to rpFVIII alone and ii) how binding of rpFVIII to ND would affect the clotting time curve in the first 20 minutes following a tail snip. The clotting tests conducted with the one stage aPTT assay (Table 1) and rotational thromboelastometry (ROTEM^®^ analyses, data not shown) against human FVIII deficient plasma *in vitro* didn’t show a significant difference between the pro-coagulant activities of rpFVIII in solution v/s that of rpFVIII bound to PS-ND. Therefore we didn’t anticipate a pronounced decrease in the overall clotting time for the rpFVIII-ND complexes injected in hemophilic mice. We were curious, however to see whether stabilizing the rpFVIII by binding to PS rich ND could affect the rate of clotting in the initial stages of bleeding after injury compared to the rpFVIII alone *in vivo*. To undertake this we carried out this first study that provided additional insight into the role of FVIII membrane binding for its function in vivo.

In blood, FVIII circulates in a tight complex with vWF (Kd ~0.2–0.5 nM) [48,49]. The high-affinity, non-covalent association of FVIII with vWF protects FVIII from otherwise rapid clearance [50,51]. The interaction between FVIII and vWF has been broadly localized to the FVIII-LC and the vWF-D’D3 domain [52–55]. Recently, the FVIII-C1 domain has been characterized as the primary binding site for the D’D3-vWF domain, the remaining C2 and A3 domains of the FVIII-LC playing an ancillary role [56]. The strong interaction between FVIII and vWF suggests that if FVIII-ND complexes are introduced in FVIII deficient blood *in vivo*, the vWF can pull the rpFVIII from the ND membrane into a more stable FVIII-vWF complex. *In vitro*, all forms of FVIII including the FVIII-LC have been shown to bind to PS rich membranes (vesicles, monolayers and nanotubes) with high affinity (Kd ~1–2 nM) [57,58]. Membrane binding has also been suggested to stabilize FVIII *in vitro* and enhance its pro-coagulant activity [59,60]. Therefore one of the advantages of FVIII binding to PS rich membranes is that the ND offer protection to FVIII from clearance, before the assembly of the FVIII-vWF complex [61,62]. This hypothesis is sustained by the higher affinity of the rpFVIII for the PS membrane (Kd ~2–6 nM) compared to FVIII affinity to the low-density lipoprotein receptor related protein (LPR) (Kd ~6–18 nM) responsible for FVIII catabolism [63,64].

There is no direct evidence that FVIII binding to artificial PS rich phospholipid (PL) membranes competes with the formation of the FVIII-vWF complex *in vitro*. This is supported by studies showing that the FVIII-vWF interaction has a Kd ~0.5 nm, which is lower than the FVIII-PL interaction Kd ~2–6 nM [56,63,65,66]. From the presented work and the results published in [56] it is conceivable that the vWF can bind to the FVIII-ND complexes through the C1-A3 interface. In this case the formation of FVIII-vWF complexes on the PS-rich ND surface will be possible if the FVIII-C1 domain is accessible to the D’D3 domain of the vWF and only the FVIII-C2 domain binds to the ND membrane. The vWF can detach from the vWF-FVIII-ND complex upon initiation of the coagulation process, leaving the FVIIIa bound to the PS rich ND accessible to the FIXa, thus forming the intrinsic tenase complex [67]. It has been shown that once the FVIII is activated, the binding affinity of FVIIIa to a PS rich membrane is significantly increased, especially once the FVIIIa-FIXa complex is formed [49]. These data support the hypothesis that the FVIIIa might remain bound to the PS rich ND surface to form the FVIIIa-FIXa complex. It is important to keep in mind that all binding constants for the FVIII complexes with PL, vWF and the LPR receptor have been determined only for the human, not the porcine FVIII form.

Another pathway that doesn’t require the presence of vWF and the formation of the FVIII-vWF complex can be also considered, based on the observed ability of the ND in this study to stabilize rpFVIII both *in vitro* (prior to the infusion) and *in vivo* (after injection) [12]. If this is the case, membrane-bound FVIII introduced in the blood stream will not bind to the vWF but rather circulate in the form of FVIII-ND complexes bypassing the FVIII-vWF complex step altogether. The FVIII-ND complexes will bind FIXa upon activation of FVIIIa on the PS rich ND surface and initiation of coagulation. This hypothesis is supported by previous studies showing that administration of FVIII bound to PS-rich liposomes compensates for a lack of vWF and restores normal clotting in a mouse model [66].

Based on our results, we cannot determine whether the rpFVIII remains bound to ND in the blood stream or detaches from ND to bind to the vWF or to the activated platelet surface upon initiation of coagulation. From our *in vivo* experiments, it is also not clear if the intrinsic tenase complexes are formed directly on the ND or on the activated platelets’ surface. As the ND employed in this study are significantly more potent than the activated platelet membrane due to their higher PS content (80% compared to 30%) and can bind FVIII molecules to both sites of the ND membrane, we could consider that they will be favored for the FVIIIa-FIXa complex assembly. However the possibility that the FVIII detaches from the ND to bind to the vWF or the activated platelet membrane surface after proteolytic activation should be also considered. In this case the ND would be seen more as a carrier of FVIII, being of similar size as the protein and capable of carrying more than one molecule FVIII. Finally the ND carrying the membrane-bound FVIII can fuse with the activated platelet surface before or after the coagulation process is initiated. To fully understand the functional aspects and mechanism of how binding to ND improves FVIII the clotting in FVIII deficient (FVIII-KO) mice, additional experiments based on our observation and experimental design should be further carried out. The competition between the FVIII binding to the ND and to the vWF, as well as the sequence of the FVIIIa-FIXa complex assembly should be characterized further *in vitro*.

Finally, our results do not show a decrease in the immunogenicity of rpFVIII bound to a PS rich ND, as has been reported for rhFVIII bound to PS rich liposomes [61]. To confirm this finding, as well as the mechanism of the increase in pro-coagulant efficiency of FVIII when bound to ND in FVIII-KO mice, we will repeat our experiments with a larger sample size and carry out additional functional tests *in vitro*.

Understanding the mechanism of how the FVIII-ND complex functions *in vivo* is an important step towards improvement of therapeutic FVIII for Hemophilia A treatment. Introducing rpFVIII-ND has the added advantage that the ND are similar to the lipoproteins circulating in blood that have been suggested to hold functional tenase complexes *in vivo* [49]. Thus by combining genetic modification of FVIII with ND assembly (PS content and type of MSP) the rpFVIII pro-coagulant activity, half-life and immunogenicity can be modulated using rpFVIII-ND complexes. This can be easily accomplished due to the established expression and purification of FVIII and ND assembly protocols [20,32].

## Conclusion

The presented results support that binding of FVIII to PS rich ND increases its stability and pro-coagulant activity in a mouse model of Hemophilia A (FVIII-KO). The improvement of the rpFVIII pro-coagulant properties when preassembled in rpFVIII-ND complexes can be attributed to the shielding effect of the ND for the rpFVIII binding site to the LPR. The stabilizing effect of the ND coupled with their pro-coagulant effect could have the added benefit of reducing the amount of FVIII required for preventing bleeding episodes in Hemophilia A patients and thus lower the cost of treatment.

## Figures and Tables

**Table 1:** Activity of pFVIII and pFVIII bound to ND (pFVIII-ND) as measured with the one stage aPTT clotting assay in FVIII deficient human plasma. The pFVIII and ND were mixed in 10 times excess of ND to account that all pFVIII is bound to the ND. The ND alone didn’t have a procoagulant effect in FVIII deficient plasma.

Samples	Clotting time	Activity	Activity
	Diluted (sec)	Diluted (U/ml)	Total (U/ml)
rpFVIII	58.1	0.19	1530
rpFVIII-ND	57.7	0.2	1777
